# INHBA–S100A16 dysregulation enables a non-invasive molecular stratification platform for rapid detection of oral squamous cell carcinoma: results from a large diagnostic case-control study

**DOI:** 10.1186/s40364-026-00963-7

**Published:** 2026-06-23

**Authors:** Muy-Teck Teh, Ranjitkumar Patil, Satyajit Ashok Tekade, Deepika Mishra, Akhilanand Chaurasia, Ahmad Waseem

**Affiliations:** 1 Centre for Oral Immunobiology & Regenerative Medicine, Institute of Dentistry, Barts & The London School of Medicine and Dentistry, Queen Mary University of London, The Blizard Building, 4, Newark Street, London, E1 2AT UK; 2 Department of Oral Medicine & Radiology, King George’s Medical University, Lucknow, Uttar Pradesh India; 3 Department of Oral & Maxillofacial Pathology, Modern Dental College & Research Centre, Indore, Madhya Pradesh India; 4 Department of Oral Pathology & Microbiology, Centre of Dental Education & Research, All India Institute of Medical Sciences, New Delhi, India

**Keywords:** qMIDS, INHBA, S100A16, YAP1, POLR2A, Oral cancer, Head and neck cancer, Early oral cancer biomarkers, Squamous cell carcinoma, mRNA, Brush biopsy, RT-qPCR, Non-invasive, Oral cancer screening, Early detection

## Abstract

**Background:**

Early diagnosis of oral squamous cell carcinoma (OSCC) is critical, yet most oral potentially malignant disorders (OPMDs) are benign, and patients frequently undergo unnecessary invasive scalpel biopsies, creating diagnostic delays and patient harm. A rapid, non-invasive molecular diagnostic tool could improve early detection and reduce unnecessary procedures. This study aims to determine whether a previously validated microbiopsy-based multigene assay (qMIDS^V2^) could be adapted into a rapid, non-invasive brush biopsy test (qMIDS^V3^) for accurate OSCC detection. A prespecified hypothesis was that qMIDS^V3^ could distinguish OSCC from low-risk OPMD and contralateral normal mucosa with high diagnostic sensitivity.

**Methods:**

This prospective diagnostic case-control study validated a multigene mRNA test (qMIDS^V3^) for OSCC detection using 1090 oral brush biopsies from 545 patients. Each patient provided paired brush biopsies from oral lesion and contralateral non-lesion mucosa, including OSCC (*n* = 443), oral leukoplakia (OL; *n* = 63), and oral lichen planus (OLP; *n* = 39). qPCR quantified mRNA levels of four genes (*INHBA*,* S100A16*,* YAP1*,* POLR2A*) from each brush biopsy, and an algorithm generated a malignancy index for cancer risk stratification.

**Results:**

qMIDS^V3^ distinguished OSCC from OL and OLP with AUC 0.975, sensitivity 95.7%, specificity 95.1%, and overall accuracy 95.5%. False-positive and false-negative rates were 4.9% and 4.3%, demonstrating high specificity for detecting malignant cells rather than premalignant or inflammatory lesions.

**Conclusions:**

Our findings highlight the clinical utility of qMIDS^V3^ as a rapid case-finding or triage test, potentially sparing over 90% of low-risk OPMD patients from unnecessary invasive tissue biopsies while accurately identifying OSCC cases. Non-invasive brush biopsy allows safe, repeatable sampling for long-term surveillance with minimal patient harm and enhancing early oral cancer detection. By reducing unnecessary scalpel biopsies and specialty referrals, qMIDS^V3^ provides a molecularly guided and rapid triage pathway.

**Supplementary Information:**

The online version contains supplementary material available at 10.1186/s40364-026-00963-7.

## Background

In 2023, global oral cancer (lip and oral cavity; ICD10 C00-06) incidence reached an estimated 422,000 new cases, a 146% increase since 1990 [1], with Asia accounting for over 66%, followed by Europe (16%) and North America (8%) [2]. Despite advances in care, ~ 229,000 deaths occurred in 2023, a 134% increase since 1990, and 5-year survival has stagnated at ~ 50% for decades [1]. These figures exclude cancers of the nasopharynx, oropharynx, larynx and oesophagus. A recent analysis highlights widening regional and gender disparities, particularly in low- and middle-SDI regions and among women [3], consistent with Global Burden of Disease data, where lip and oral cancer ranked 3^rd^ for females (+ 54.7%) and 9^th^ for males (+ 29.2%) in global premature mortality risks from birth to age 70 between 2000 and 2023 [4].

Oral squamous cell carcinoma (OSCC), the major subtype of head and neck cancer, is often preceded by oral potentially malignant disorders (OPMDs), a heterogeneous group of lesions with variable malignant transformation risk [5–9]. Common subtypes include oral leukoplakia (OL), oral submucous fibrosis (OSF), oral lichen planus (OLP), oral erythroplakia (OE), and proliferative verrucous leukoplakia (PVL), alongside rarer conditions such as dyskeratosis congenita, actinic cheilitis, chronic hyperplastic candidosis, oral lichenoid lesions (OLL), and graft-vs-host disease–related lesions [10]. Globally, OPMD prevalence is 4.47%, ranging from 10.54% in Asia to 0.11% in North America [11]. A recent meta-analysis reported an overall malignant transformation rate of 7.9%, ranging from 49.5% in PVL, 33.1% in OE, 9.5% in OL, 5.2% in OSF, 3.8% in OLL to 1.4% in OLP [12].

Early OSCC diagnosis is critical, as advanced and recurrent disease offers limited therapeutic options. Although OPMDs provide an opportunity for early detection [5–9], no current adjunctive test—vital staining, light-based imaging, oral cytology, or saliva/blood biomarkers—can replace surgical biopsy with histopathology [13]. Owing to the invasiveness of surgical scalpel biopsy coupled with time-consuming, subjective and ambiguous diagnostic results of histological findings [7, 8, 14–16] there is great hesitancy in subjecting OPMD patients to invasive scalpel biopsy and patient acceptability is low, leading to missed opportunity and underdiagnosis [17].

As there is currently no standard quantitative method for risk stratification in OPMDs, most if not all OPMD patients are either put on regular surveillance or are biopsied and put on a variable period of review [5–7, 9, 15]. The invasiveness of scalpel biopsy often discourages both clinicians and patients from undergoing repeat procedures, delaying timely OSCC detection [18]. This creates unnecessary healthcare burden for low-risk individuals (> 90%) while risking late detection in the high-risk minority (< 10%) [19]. A non-invasive, site-specific, repeatable tool is urgently needed for long-term surveillance and timely OSCC detection. Early diagnosis and timely initiation of treatment enhance the effectiveness of cancer management, alleviate pressure on healthcare systems, and substantially improve the likelihood of favourable clinical outcomes [20].

We previously developed a multi-gene mRNA diagnostic platform, the quantitative Malignancy Index Diagnostic System (qMIDS), enabling oral cancer diagnosis using minimally invasive 1 mm³ microbiopsy. The initial version, qMIDS^V1^, was validated on cohorts from the UK and Norway [21], and subsequently on Chinese samples [22]. The enhanced version qMIDS^V2^ has been validated through an international multi-cohort study involving 535 tissue specimens from geographically and ethnically diverse patient cohorts (UK, India, and China) [23]. The qMIDS platform quantifies mRNA expression levels of 16 genes and applies a proprietary algorithm to generate a malignancy index for OSCC detection and risk stratification of OPMDs [21, 23]. However, because OPMDs often span large or multifocal areas, relying on a single 1 mm³ tissue biopsy risks sampling error. Brush biopsy offers a non-invasive alternative that samples a broader epithelial area and it has been shown to be a reliable tool for oral cancer screening [24, 25]. The primary endpoint of this study is to evaluate the diagnostic performance of qMIDS for non-invasive OSCC detection using brush biopsy specimens.

## Methods & Materials

### Clinical samples

**Fig. 1:**
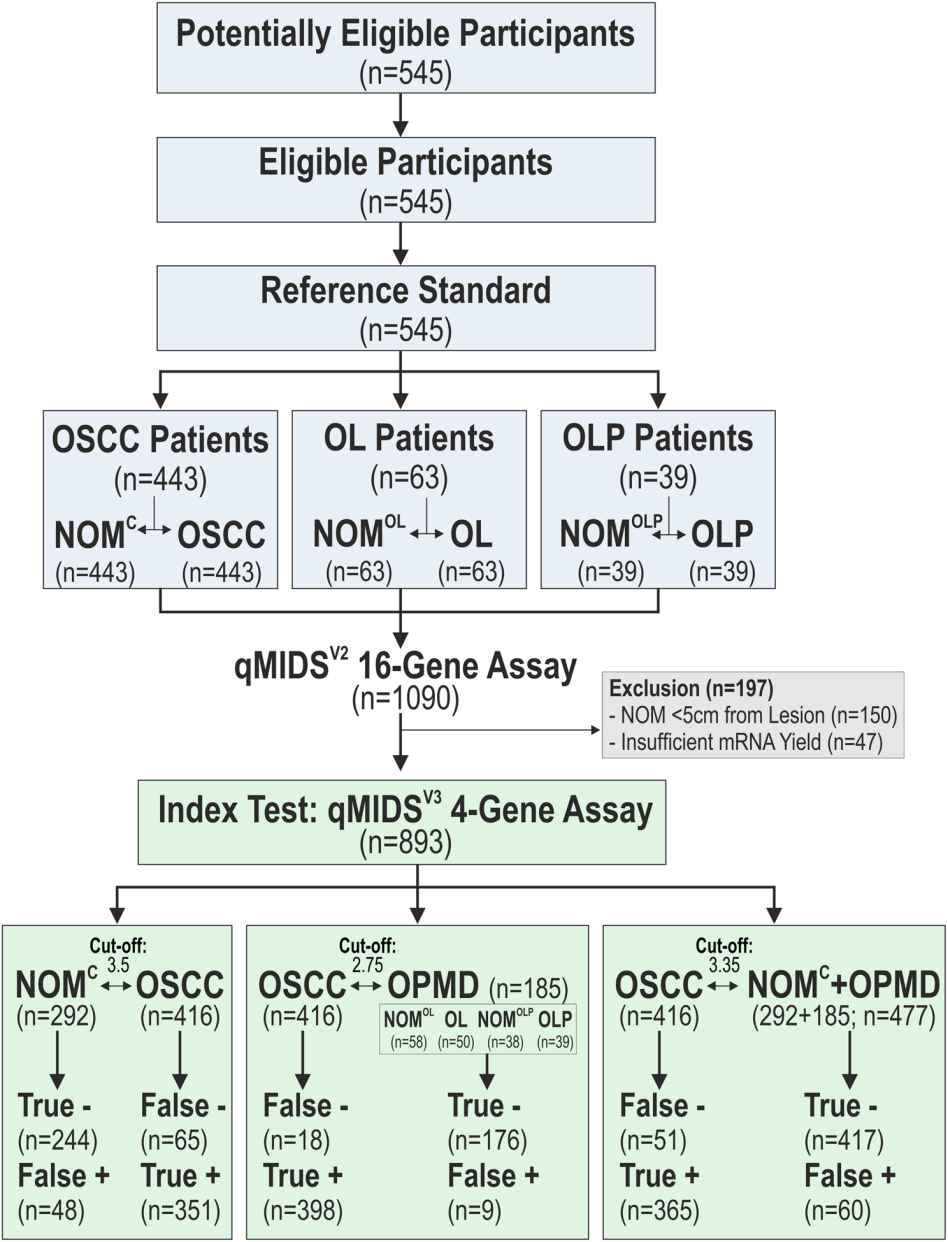
STARD flow diagram depicting participant inclusion, sample processing, and diagnostic classification. A total of 545 eligible participants underwent reference-standard histopathological diagnosis and provided paired brush biopsy samples across oral squamous cell carcinoma (OSCC; *n* = 443), oral leukoplakia (OL; *n* = 63), and oral lichen planus (OLP; *n* = 39) groups. All 1,090 brush biopsy samples were analysed using the qMIDS^V2^ 16-gene assay, with 197 samples excluded for contralateral sampling of less than 5 cm from the lesion (*n* = 150) or insufficient mRNA yield (*n* = 47). All eligible samples (*n* = 893) proceeded to the qMIDS^V3^ 4-gene assay (Index Test). Diagnostic outcomes using optimized cut-offs (3.5 for NOMᶜ vs. OSCC, 2.75 for OSCC vs. OPMD and 3.35 for OSCC vs. NOM^c^+OPMD) are shown, including true-positive, false-positive, true-negative, and false-negative results, respectively

This investigation was conducted as a diagnostic case-control validation study, in which cases (OSCC) and controls (OL, OLP, and contralateral normal mucosa) were selected based on confirmed histopathological diagnosis rather than prospective outcome follow-up, consistent with STARD definitions for diagnostic accuracy research. A STARD patient/sample flow diagram is shown in Fig. 1. The study was approved by the relevant research ethics committees (QMERC20.142, QMERC0866, 104th ECM IIA/P18) and the study was conducted in accordance with the Declaration of Helsinki. Brush biopsies were collected from adults ≥ 18 years between October 2021 and June 2024 with informed consent and corresponding clinico-histopathological reports. Eligible participants had confirmed OSCC, OL (hyperkeratosis and/or epithelial hyperplasia without dysplasia), or OLP within 6 months of sampling. Exclusions included concurrent malignancies, autoimmune or chronic inflammatory disease, inaccurate contralateral sampling (< 5 cm from the lesion), or insufficient RNA yield for SYBR green detection of at least 6 target genes in the initial qMIDS^V2^ 16-gene assay [23] or 2 target genes in the subsequent qMIDS^V3^ 4-gene assay and the 2 reference genes by RT-qPCR quantification. The primary endpoint of this study was to determine the diagnostic performance of the qMIDS^V3^ brush biopsy assay for detecting OSCC, measured by its ability to discriminate OSCC from low-risk OPMDs (OL without dysplasia and OLP) and/or contralateral normal mucosa, as quantified by the area under the receiver operating characteristic curve (AUC), sensitivity, and specificity.

**Fig. 2:**
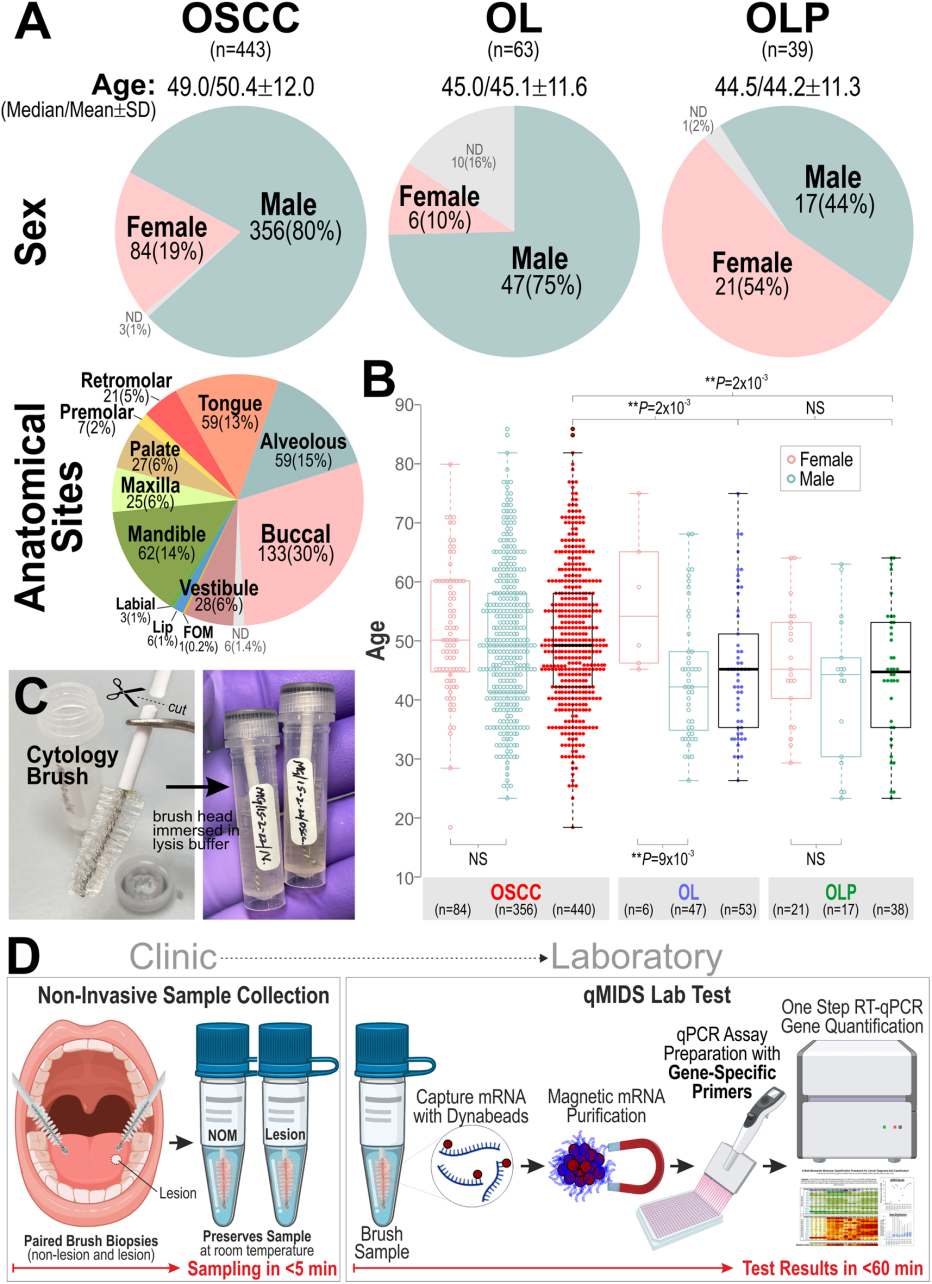
Patient demographics, oral cytology brush sampling method and qMIDS workflow. **A**, Patient age and sex demographics of oral squamous cell carcinoma (OSCC), oral leukoplakia (OL), oral lichen planus (OLP) and anatomical sites of OSCC cohort. **B**, Patient age and sex distribution for each OSCC, OL and OLP cohorts plotted as Beeswarm scattered box-whisker dot plots (box horizontal lines represent median and 25–75% percentiles, whiskers represent lowest and highest values, outliers are beyond the whiskers) showing age distribution of males and females in each cohort and corresponding unpaired *t*-test analyses *P*-values are indicated within the figure. NS, not significant. **C**, Cytology brush sampling procedure used in this study. **D**, An overview of qMIDS test workflow. At the clinic, paired non-invasive brush biopsy samples were taken from each patient, one from non-lesional oral mucosa (NOM) and another from the oral lesion (test site) using cytological brushes shown in C. Sampling procedure generally took less than 5 min. Brush heads were detached from the stem and immediately inserted the brush head into sample tubes containing a storage buffer that digests and stabilises RNA at room temperature (Supplementary Figure S1). At the diagnostic laboratory, mRNA was purified using Dynabeads magnetic system and gene-specific primers were used in a 1-step RT-qPCR system for mRNA quantification in a qPCR instrument. An algorithm converts the qPCR data into qMIDS malignancy index for oral cancer detection and OPMD risk stratification [21, 23]. The entire qMIDS assay takes < 60 min to generate test results

Sterile cytological brushes (#LMCB000; Luck Medical Consumables Co. Ltd., Jiangsu, China) were used for non-invasive sampling of paired lesion and contralateral mucosa before tissue biopsy. Brush head was rotated 10 times to collect full thickness mucosal epithelial cells. This technique does not compromise downstream histopathological assessment [26]. Brush head was then detached from the stem and immediately inserted into a 2 mL screw-cap tube containing 0.8 mL a lysis buffer (Dynabeads mRNA Direct Kit, #61012, ThermoFisher) with 0.2% proteinase K (#03115828001, Roche) and stored at 4 °C. Stability testing showed that mRNA preserved in the lysis buffer was stable for at least 6, 4 and 1 month at respective temperate (Supplementary Figure S1A). qMIDS index remained stable throughout the 15-month period when kept at 4 °C, 10 months at room temperature and 3 months at 32 °C (Supplementary Figure S1B). Sample lysate (200 µL) was used for mRNA extraction using Dynabeads mRNA Direct kit [21, 22]. All samples were coded, link-anonymised and tested blindly. Patient demographic data are summarised in Fig. 2A-B.

### The qMIDS assay protocol

The qMIDS assay was performed as described previously [23]. In brief, one-step RT-qPCR was conducted using qPCRBIO SyGreen 1-Step Go (PCR Biosystems). Initial analyses used the qMIDS^V2^ 16-gene format, quantifying 14 target genes (*HOXA7*, *CENPA*, *NEK2*, *DNMT1*, *INHBA*, *FOXM1*, *TOP2A*, *BIRC5*, *MMP13*, *CXCL8*, *IVL*, *NR3C1*, *CBX7* and *S100A16*) and two reference genes (*YAP1*,* POLR2A*) on a MIQE-compliant [27] protocol using a 384-well Roche LightCycler 480 II system. Subsequently, all qualifying samples were re-assayed using the dedicated qMIDS^V3^ four-gene panel (*INHBA, S100A16, YAP1, POLR2A*; Supplementary Figure S2). Thermocycling begins with 45 °C for 10 min followed by 95 °C for 30 s prior to 45 cycles of amplification at 95 °C for 1 s, 60 °C for 1 s, 72 °C for 1 s, 78 °C for 1 s (data acquisition). To maximise primer specificity, we included a ‘touch-down’ annealing temperature intervention (prior to the amplification step) with a starting temperature at 66 °C followed by a stepwise reduction of 0.6 °C/cycle; 8 cycles. Relative mRNA abundance was calculated using the Roche LightCycler Software which is based on the second derivative maximum algorithm method [28]. Primer sequences, assay specificity, and metadata have been published [21, 23, 29]. Expression data were exported to Microsoft Excel for computation of qMIDS malignancy indices (MI) using the established algorithm (see below). No-template controls were included by processing lysates without biopsy material.

### The qMIDS algorithm

$$MI = {\sum\limits_{i = 1}^n {\left| {Lo{g_2}\left[ {{{T\left( {{T_{nm}}} \right)} \over {T_{n}\left( {{T_m}} \right)}}} \right]} \right|} _i} \cdot Lo{g_2}\left[ {{Q_4}\left( {{Q_3} - {Q_1}} \right)} \right]$$

The qMIDS algorithm [21, 23, 29] above was developed for computing and translating multigene expression signatures from sample into a qMIDS Malignancy Index (MI) for risk stratification. *T* represents the relative expression of each target gene (normalised against 2 reference genes); *T*_*n*_ represents the sum of *n* number of target genes measured within in each sample; *T*_*m*_ represents a median value of *T* derived from a panel of control samples (normal oral mucosa) within the study cohort; *T*_*nm*_ represents the sum of the *n* number of *T*_*m*_ values. *Q*_*1*_, *Q*_*3*_ and *Q*_*4*_ represent the first (25%), third (75%) and forth (100%) rank quartile of the *x* number of target gene absolute fractional Log_2_ ratio (|*Log*_*2*_ *[T(T*_*nm*_*)/T*_*m*_*(T*_*n*_*)]*|) distribution values within each sample. MI values were then proportionately calculated to fit a linear scale.

### Statistical analysis

Statistical t-test (*P*-value; Microsoft Excel) test were used for differential analysis between two groups of data. Receiver operating characteristics (ROC) curves were generated to obtain area under the ROC curves (AUC) to assess diagnostic efficiency (performed using Pivot-table method within Microsoft Excel and DeLong test using the pROC package [30] in R version 4.5.2; The R Foundation for Statistical Computing). Diagnostic test performance based on cut-off values were calculated using a Diagnostic Test Calculator [31]. Scattered box-whisker dot plots were created in R using the Beeswarm package (https://github.com/aroneklund/beeswarm) [32].

## Results

INHBA and S100A16 as key OSCC biomarkers in oral brush biopsies.

We investigated the feasibility of applying oral brush biopsies to the qMIDS^V2^ assay, previously validated on tissue specimens [23]. The analysis included 1,090 paired lesion and contralateral normal oral mucosa (NOM^C^, NOM^L^, or NOM^P^) samples from 545 patients across OSCC (*n* = 443), OL (*n* = 63), and OLP (*n* = 39) respective cohorts (Fig. 1). Expression of 16 genes was quantified by RT-qPCR, and a proprietary algorithm generated the qMIDS malignancy index for cancer risk assessment [21, 23].

**Fig. 3:**
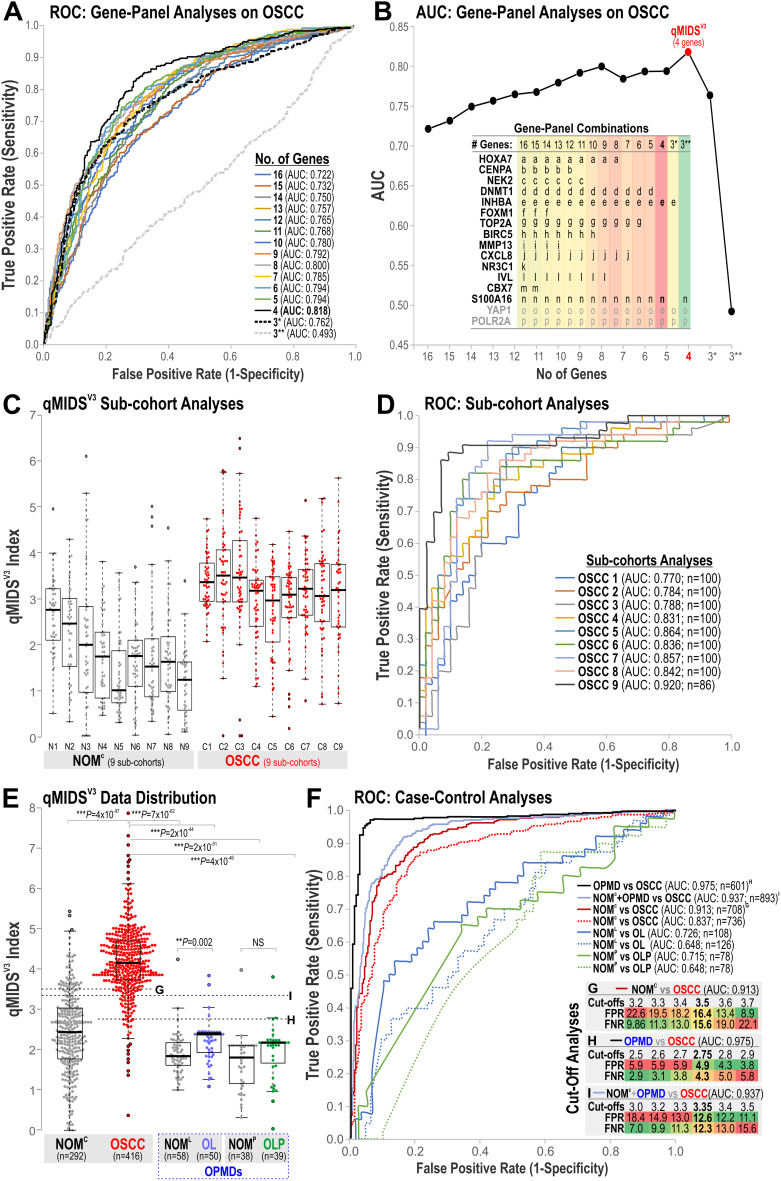
qMIDS test performance characterisation and optimisation for oral brush biopsy samples. **A**, Gene-panel ROC analyses on OSCC cohort to investigate qMIDS test performances across gene combinations from 16 down to 3 genes (1 target and 2 reference genes) with corresponding AUC values listed within the figure. **B**, AUC data from panel A is plotted against the number of genes with an inset table showing corresponding gene combinations. The 16-gene panel consists of 14 target genes and 2 internal reference genes. The 4-gene combination (named qMIDS^V3^) demonstrated the highest discriminatory power (AUC = 0.818) on gene expression data obtained from 16-gene assay format. **C**, qMIDS^V3^ OSCC sub-cohort analyses. Contralateral normal oral mucosa (NOM^C^) and OSCC cohort samples were subdivided into 9 sub-cohort as indicated (N1-9 and C1-9). Datapoints representing qMIDS^V3^ index values obtained from individual samples were plotted as Beeswarm scattered box-whisker dot plots (box horizontal lines represent median and 25–75% percentiles, whiskers represent lowest and highest values, outliers are beyond the whiskers). **D**, ROC analyses on each OSCC sub-cohort pairs (from C) with their corresponding AUC values and n sample size indicated within the figure. **E**, qMIDS^V3^ Beeswarm scattered box-whisker dot plots showing qMIDS Malignancy Index data distribution obtained from 4-gene assay for OSCC, OL and OLP cohorts, each with corresponding contralateral NOM^C, L or P^ samples, respectively. Paired *t*-test analyses *P*-values are indicated within the figure. NS, not significant. Dotted lines represent the optimal cut-off values to differentiate between corresponding cohorts (see panel G, H and I for cut-off analyses). **F**, ROC case-control analyses with corresponding AUCs and sample size as shown within the figure. Dotted lines represent 4-gene data extracted from initial 16-gene assays, whilst solid line represents data from repeat 4-gene assays performed directly from brush samples. The reduction in sample sizes in the repeat experiment using 4-gene assay compared with the original 16-gene assay was due to some samples failed mRNA QC inclusion criteria. **G**, Cut-off analyses for NOM^C^ vs. OSCC. **H**, Cut-off analyses for OPMDs (consists of pooled data from NOM^L^, OL, NOM^P^ and OLP cohorts shown in panel E – dotted blue box) vs. OSCC. **I**, Cut-off analyses for NOM^c^+OPMDs vs. OSCC with corresponding false-positive rate (FPR) and false-negative rate (FNR) at each of the cut-off values analysed. Colour heatmap was applied to FPR and FNR values to indicate high (red) to low (green)

As brush cytology provides less cellular material than tissue biopsy, several genes in the 16-gene assay were undetectable. To refine the assay, we performed gene-panel titration using sequential gene removal and ROC/AUC comparison (Fig. 3A–B). To quantify the contribution of each gene to overall classifier performance, we systematically evaluated all possible single-gene omissions using a stepwise titration strategy. Starting with the full 16-gene panel we removed each gene individually and recalculated the corresponding AUC values. The gene whose removal resulted in the smallest decrease in AUC was considered the least contributory and therefore designated as dispensable. Using this criterion, *NR3C1* emerged as the first gene to be removed, followed sequentially by *CBX7*,* FOXM1*,* MMP13*,* CENPA*,* NEK2*,* BIRC5*,* IVL*,* HOXA7*,* CXCL8*,* TOP2A* and *DNMT1*. The complete elimination sequence and its effect on AUC are presented in Fig. 3B, where the inset table illustrates all gene-panel combinations assessed during this iterative reduction process. A 4-gene panel comprising two target genes (*INHBA* and *S100A16*) and two reference genes (*YAP1* and *POLR2A*) demonstrated the strongest discrimination between NOM^C^ and OSCC (AUC of 0.818), establishing the qMIDS^V3^ configuration.

*INHBA* was significantly upregulated in all three lesion types compared with their paired NOM controls (Supplementary Figure S3A), whereas *S100A16* was significantly downregulated only in OSCC (Supplementary Figure S3B). Together, *INHBA* and *S100A16* contributed co-dependent signals within the qMIDS^V3^ 4-gene panel, and exclusion of either reduced AUCs to 0.762 (*INHBA*) or 0.493 (*S100A16*) (Fig. 3A–B). We have previously observed similar phenomenon in another study investigating differentially expressed genes in OSCC and also in nasopharyngeal cancer [23, 29] that differential expression at individual gene level may not present significant discriminatory power but it can be significantly amplified when combined with other genes using the qMIDS algorithm.

### Gender and age factors on qMIDS^V3^ test performance

As *INHBA* has been implicated in regulating the female reproductive system [33], we evaluated potential gender effects on qMIDS^V3^ performance. Stratified NOM^C^ and OSCC cohorts showed no gender-related differences in gene expression or assay performance (female, *n* = 84, AUC = 0.819; male, *n* = 356, AUC = 0.822) (Supplementary Figure S4A-B). Similarly, performance also remained consistent across all age groups (< 40, *n* = 95; 41–50, *n* = 146; 51–60, *n* = 111 and > 60 years, *n* = 88) (Supplementary Figure S4C-D).

### qMIDS^V3^ sub-cohort analyses

Over a 32-month period, 886 OSCC samples were collected. To assess potential heterogeneity, paired NOM^C^ (*n* = 443) and OSCC (*n* = 443) samples were divided into nine chronological sub-cohorts (N1–9 and C1–9; Fig. 3C–D). Variability was observed in NOM^C^ sub-cohorts N1–3, while N4–9 and all OSCC sub-cohorts were homogeneous (Fig. 3C).

To further evaluate diagnostic consistency, ROC analyses were performed for each matched NOM^C^–OSCC sub-cohort (e.g., N1 vs. C1, N2 vs. C2). Early pairs (N1–3 vs. C1–3) showed lower AUCs (0.770–0.788), whereas later pairs demonstrated higher performance (0.831–0.920) (Fig. 3D). A potential confounding factor was later identified: early-stage NOM^C^ samples were collected < 5 cm away from the tumour site, contributing to the observed heterogeneity in N1–3. Consequently, sub-cohorts N1–3 were excluded from subsequent analyses.

Because the initial gene-titration analyses was based on 16-gene assay format, we re-tested all qualifying brush biopsy samples with the dedicated 4-gene qMIDS^V3^ assay (Supplementary Figure S2A-B). Samples with insufficient mRNA were excluded in accordance with predefined exclusion criteria. The 4-gene assay showed a segregation between NOM^C^ (*n* = 292) and OSCC (*n* = 416) with an AUC of 0.913 (Unpaired *t*-test, *P* = 4 × 10^–97^; Fig. 3E-F).

### qMIDS^V3^ specifically detects OSCC but not OL or OLP

To evaluate if qMIDS^V3^ could differentiate between OSCC and oral premalignant or inflammatory conditions, we further tested oral leukoplakia (OL) or oral lichen planus (OLP) and their paired contralateral normal mucosa samples (NOM^L^ or NOM^P^) (Fig. 3E). While NOM^L^ samples showed significant segregation from OL (Paired *t-*test, *P* = 0.002), the qMIDS^V3^ index values remained lower than those observed in OSCC, potentially reflecting the modest malignant transformation risk associated with OL without dysplasia [34] In contrast, NOM^P^ and OLP samples did not differ significantly, consistent with the low malignant transformation potential of OLP [35].

Overall, qMIDS^V3^ index values were significantly higher in OSCC than in NOM, OL, or OLP. This was further supported by ROC analyses that the dedicated 4-gene assay (solid lines, Fig. 3F) outperformed the 16-gene extracted dataset (dotted lines) yielding AUCs of 0.913 vs. 0.873 (OSCC vs. NOM), 0.762 vs. 0.648 (OL), and 0.715 vs. 0.648 (OLP).

To define the optimal cut-off for distinguishing NOM^C^ from OSCC, we evaluated false-positive (FPR) and false-negative (FNR) rates across a range of thresholds (Fig. 3G); 3.5 provided the best balance (FPR 16.4%, FNR 15.6%). When OPMD cohorts (NOM^L^, OL, NOM^P^, OLP) were compared with OSCC, the assay achieved an AUC of 0.975 (Fig. 3F), with an optimal cut-off at 2.75 yielding an FPR of 4.9% and an FNR of 4.3% (Fig. 3H). When NOM^c^ were added to OPMD and compared with OSCC, the assay achieved an AUC of 0.937 with an optimal cut-off at 3.35 yielding an FPR of 12.6% and an FNR of 12.3% (Fig. 3I).

### qMIDS test performance comparison between tissue vs. brush biopsy

**Fig. 4:**
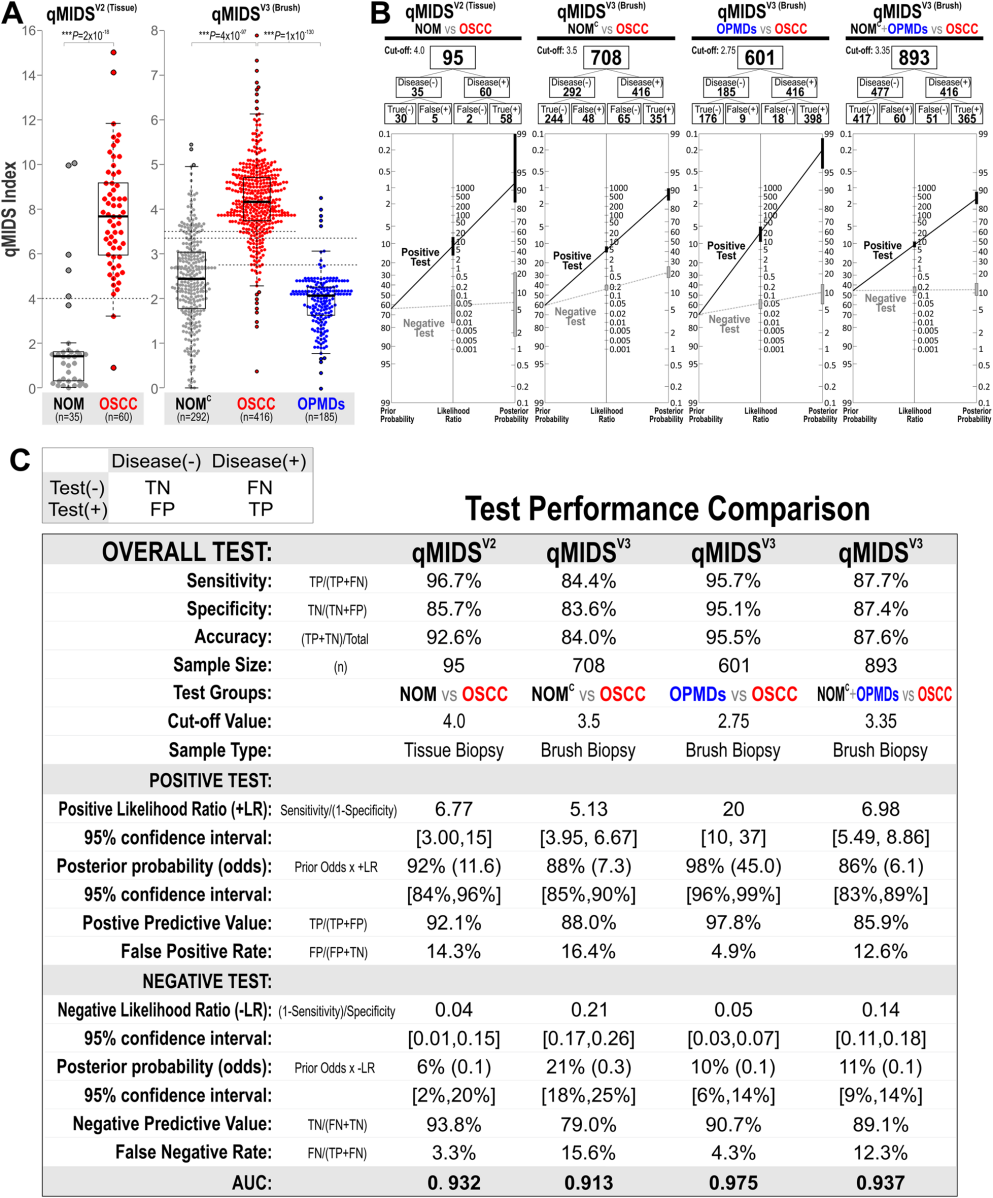
qMIDS test performance comparison between microbiopsy tissue vs. oral brush biopsy samples. **A**, qMIDS^V2^ tissue biopsy data extracted from our previously published geographically matched patient’s cohort [23] with margin NOM or core OSCC tissues, in comparison with qMIDS^V3^ oral brush biopsy data (reproduced from Fig. 2E). Dotted lines indicate corresponding optimum cut-off values. OPMD cohort consists of NOM^L^, OL, NOM^P^ and OLP samples. Unpaired *t*-test analyses *P*-values are indicated within the figure. **B**, Test performances for qMIDS^V2^ (16-gene assays performed on tissue biopsies) and qMIDS^V3^ (4-gene assays performed on brush biopsies) for corresponding cohort pairs were calculated based on respective best cut-off values identified from Fig. 2G-I. **C**, Test performance comparison table for qMIDS^V2^ and qMIDS^V3^. TN, true negative; FN, false negative; FP, false positive; TP, true positive; AUC, area under the curve obtained from ROC analyses

We next compared tissue-based qMIDS^V2^ with brush-based qMIDS^V3^ (Fig. 4). The qMIDS^V2^ tissue biopsy data were extracted from our previously published, geographically matched patient cohort, which compared non-lesional oral mucosa (NOM, *n* = 35) to core OSCC tumour (*n* = 60) microbiopsy tissues [23]. Using paired NOM^C^-OSCC brush biopsies, qMIDS^V3^ achieved an AUC of 0.913 (*n* = 708), with sensitivity, specificity, and overall accuracy each at 84%, and predictive values of 88% (PPV) and 79% (NPV). These metrics were slightly lower than qMIDS^V2^ (sensitivity 97%, specificity 86%, accuracy 93%, AUC 0.932, *n* = 95; Fig. 4A).

Notably, when OSCC samples were compared with pooled OPMD samples (including NOM^L^, OL, NOM^P^, and OLP), qMIDS^V3^ performance improved markedly, achieving an AUC of 0.975 (*n* = 601), with 95.7% sensitivity, 95.1% specificity, 95.5% accuracy, and predictive values of 97.8% (PPV) and 90.7% (NPV). Furthermore, false positive and false negative rates were substantially reduced—from 16.4% to 15.6% to 4.9% and 4.3%, respectively (Fig. 4C).

When OSCC samples were compared with pooled NOM^C^ plus OPMD, qMIDS^V3^ performance was impacted slightly, achieving an AUC of 0.937 (*n* = 893), with 87.7% sensitivity, 78.4% specificity, 87.6% accuracy, and predictive values of 85.9% (PPV) and 89.1% (NPV). False positive and false negative rates were 12.6% and 12.3%, respectively (Fig. 4C).

## Discussion

This study examined the performance of a 16-gene mRNA diagnostic panel (qMIDS^V2^) for non-invasive oral cancer detection using brush biopsies. The qMIDS^V2^ test had previously been validated through an international multi-cohort diagnostic case-control study involving 535 microbiopsy tissue samples from UK, China, and India, showing consistent differential expression of the 14 target genes across geographically and ethnically diverse OSCC cases, with additional predictive value for malignant transformation in oral epithelial dysplasia [23].

In this study, the 16-gene qMIDS^V2^ panel was applied to 1,090 brush biopsy samples from 545 patients (OSCC = 443, OL = 63, OLP = 39), each providing paired lesion and contralateral mucosa. Gene-panel titration identified an optimized 4-gene set (*INHBA, S100A16, YAP1, POLR2A*) with the highest diagnostic performance, designated qMIDS^V3^. qMIDS^V3^ achieved an AUC of 0.913 for OSCC vs. NOM^C^, comparable to tissue-based qMIDS^V2^ (AUC 0.932). The slight reduction in qMIDS^V3^ (brush biopsy) performance relative to qMIDS^V2^ (tissue biopsy) may reflect field cancerisation, where molecular abnormalities extend beyond the visible tumour margin [36], may reduce discrimination between cancerous and contralateral mucosa. Furthermore, there is a possibility that dissemination of exfoliated tumour cells throughout the oral cavity may further raise background noise and reduce test specificity. In fact, the higher baseline values from NOM^c^ (contralateral normal oral mucosa sample from OSCC patients) weaken the qMIDS^V3^ performance for distinguishing OSCC from NOM^c^+OPMD (AUC 0.937; sensitivity 88%; specificity 87%). However, OSCC against OPMD alone, performance improved significantly (AUC 0.975; sensitivity 96%; specificity 95%; accuracy 96%; PPV 98%; NPV 91%; FPR 4.9%; FNR 4.3%). qMIDS^V3^ performance was unaffected by age or gender, supporting its robustness. To our knowledge, this is the first validated non-invasive assay capable of reliably distinguishing OSCC from OPMDs with such high accuracy.

*INHBA* [37, 38], *S100A16* [39] are key OSCC-associated markers involved in stromal and immune modulation. The *INHBA* gene encodes for inhibin subunit βA protein, a transforming growth factor β (TGFβ) superfamily member [33]. We have previously shown that *INHBA* mRNA was differentially upregulated in HNSCC tumour tissues from geographically and ethnically diverse patient cohorts [23, 40] and pan-cancer Kaplan-Meier survival analysis further revealed that *INHBA* predicted poor prognosis in 16 out of 21 different human cancer types, including HNSCC [40]. In contrast, *S100A16* gene has a tumour suppressive role in OSCC as *S100A16* mRNA and protein was found to be progressively downregulated from normal oral mucosa to oral dysplastic lesions and OSCC [39, 41]. Furthermore, we have previously shown that *S100A16* mRNA was differentially downregulated in HNSCC tumour tissues from geographically and ethnically diverse patient cohorts [23, 40]. In this study, combining *INHBA* and *S100A16* with the reference genes *YAP1* and *POLR2A* was essential for qMIDS^V3^ test specificity in distinguishing OSCC from OPMDs, reducing false positives in low-risk OPMD lesions.

There remains a clear need for a non-invasive, rapid, and cost-effective case-finding tool to reduce the growing burden of oral cancer on healthcare systems. In the UK, a 10-year audit reported a 450% rise in two-week-wait referrals alongside a 50% drop in cancer detection rates [42]. Subsequent audits showed that 92.5–99.5% of referred patients were cancer-free, with most (96–98%) remaining cancer-free at 5-year follow-up [43, 44]. These findings highlight substantial over-referral and inefficiency in current pathways. Implementing a rapid, non-invasive triage test such as qMIDS^V3^ could reduce unnecessary referrals [44] for > 90% of cancer-free patients and lessen the clinical and financial burden of managing OPMDs and OSCC. Furthermore, qMIDS^V3^ supports repeat brush biopsy, a critical capability for long-term surveillance, facilitating the detection of malignant transformation in OPMD patients while avoiding the need for multiple scalpel biopsies.

Recent consensus demonstrated that oral brush cytology can be a reliable and cost-effective tool for oral cancer screening [24, 25]. However, a systematic review found insufficient high-certainty evidence to support population-level screening for oral cancer or OPMD in asymptomatic adults [45]. Current efforts therefore focus on case-finding in patients with clinically suspicious lesions. Although imaging-based brush cytology has been explored as a non-invasive adjunct, these methods lack the specificity needed to distinguish OPMDs from OSCC [13, 46, 47]. In this study, we show that non-invasive brush biopsy combined with rapid (≤ 60 min) mRNA quantification provides a molecular solution to this gap. Using 1,090 samples, qMIDS^V3^ demonstrated high accuracy (AUC 0.975) in detecting OSCC and differentiating it from common non-dysplastic OL and OLP. This level of specificity is essential for a triage tool that identifies the minority (< 10%) requiring biopsy while reducing unnecessary harmful biopsy procedures, support a de-escalation strategy for low-risk lesions, enable repeatable non-invasive surveillance required for early cancer detection, ease patient anxiety, and potentially increase cancer detection in those who do not currently receive a biopsy referral or those who refused tissue biopsy.

With global OSCC incidence projected to rise by 65% by 2050 and mortality disproportionately affecting low-HDI countries, there is an urgent need for cost-effective and accessible diagnostic solutions. Early detection remains central to improving survival [48]. qMIDS^V3^ offers advantages for low-resource settings: brush sampling can be performed by trained nurses and room-temperature mRNA stability eliminates cold-chain requirements. Expanded global RT-qPCR infrastructure post-COVID-19 [49] enables scalable, rapid automatable testing, and the assay uses inexpensive, widely available consumables (< USD $10 per sample), supporting equitable deployment.

### Strengths and limitations

This diagnostic case-control study has several strengths. It includes 1,090 oral brush biopsy samples, representing one of the largest OSCC brush biopsy cohorts to date, with paired lesion and contralateral mucosa providing strong statistical power for validating diagnostic accuracy. Gene-panel titration identified a refined four-gene qMIDS^V3^ assay, which demonstrated high performance for distinguishing OSCC from OPMDs (AUC 0.975; sensitivity 96%; specificity 95%) and remained robust across gender and age groups. However, several limitations should be noted. First, the cohort was drawn from a single regional population in Uttar Pradesh, India, which may limit broader generalizability; however, all 16 qMIDS biomarkers were previously validated across ethnically and geographically diverse cohorts from the UK, China, and two independent Indian populations (Uttar Pradesh and Karnataka) [23], supporting their wider applicability. Second, the sample sizes for OSCC, OL, and OLP were unequal and do not reflect true disease prevalence, which may affect comparative analyses. Third, the study lacked an independent healthy NOM brush biopsy cohort, limiting assessment of baseline molecular variability. Despite these constraints, the large OSCC sample size and paired contralateral design provide strong statistical power, and inclusion of OL and OLP strengthens evidence for qMIDS^V3^ specificity against non‑malignant and inflammatory conditions.

## Conclusions

This study, the largest of its kind with 1,090 oral brush biopsy samples, validated the third-generation qMIDS^V3^ assay. Using a four-gene mRNA panel (*INHBA*,* S100A16*,* YAP1*,* POLR2A*), the test enables rapid, non-invasive OSCC detection with results available within one hour. qMIDS^V3^ showed promising performance in distinguishing OSCC from non-dysplastic OPMD. Its non-invasive sampling, room-temperature stability, and rapid turnaround make qMIDS^V3^ well suited for low-resource settings. By enabling repeatable sampling and potential point-of-care triage, the assay may reduce unnecessary scalpel biopsies and support earlier identification of high-risk cases through data-driven clinical triage.

## Supplementary Information

Below is the link to the electronic supplementary material.

Supplementary Material 1

## Data Availability

The data that supports the findings of this study is available as supplementary materials.

## Abbreviations

AUC: Area under the ROC Curve
BIRC5: Baculoviral IAP repeat-containing protein 5 (Survivin)
CBX7: Chromobox 7
CENPA: Centromere protein A
CXCL8: C-X-C motif chemokine ligand 8 (IL-8)
DNMT1: DNA methyltransferase 1
FOXM1: Forkhead box M1
FNR: False-negative rate
FPR: False-positive rate
HNSCC: Head and neck squamous cell carcinoma
HOXA7: Homeobox A7
HPV: Human papilloma virus
INHBA: Inhibin subunit βA
IVL: Involucrin
MIQE: Minimum Information for publication of quantitative real-time PCR experiments
MMP13: Matrix metallopeptidase 13
mRNA: messenger RNA
NEK2: NIMA-related kinase 2
NOM: Normal oral mucosa
NPV: Negative predictive value
NR3C1: Glucocorticoid receptor
NTC: No template control
OE: Oral erythroplakia
OL: Oral leukoplakia
OLP: Oral lichen planus
OPMD: Oral potentially malignant disorder
OSCC: Oral squamous cell carcinoma
OSF: Oral submucous fibrosis
POLR2A: RNA polymerase II subunit A
PPV: Positive predictive value
PVL: Proliferative verrucous leukoplakia
qMIDS: quantitative malignancy index diagnostic system
ROC: Receiver operating characteristic curve
RT: Reverse transcription
RT-qPCR: Reverse transcription quantitative polymerase chain reaction
RUNX2: Runt-related transcription factor 2
S100A16: S100 calcium-binding protein A16
S100A8/A9: Calprotectin complex
SCC: Squamous cell carcinoma
SDI: Socio-demographic index
STARD: Standards for reporting diagnostic accuracy
TGFβ: Transforming growth factor beta
TOP2A: Topoisomerase II alpha
YAP1: Yes-associated protein 1
miR-376c: MicroRNA-376c

## References

[1] Collaborators GBDC. The global, regional, and national burden of cancer, 1990–2023, with forecasts to 2050: a systematic analysis for the Global Burden of Disease Study 2023. Lancet. 2025;406(10512):1565–86. 41015051 10.1016/S0140-6736(25)01635-6 PMC12687902

[2] Sung H, Ferlay J, Siegel RL, Laversanne M, Soerjomataram I, Jemal A, Bray F. Global Cancer Statistics 2020: GLOBOCAN Estimates of Incidence and Mortality Worldwide for 36 Cancers in 185 Countries. CA Cancer J Clin. 2021;71(3):209–49. 33538338 10.3322/caac.21660

[3] Hu Q, Lv S, Wang X, Pan P, Gong W, Mei J. Global burden and future trends of head and neck cancer: a deep learning-based analysis (1980–2030). PLoS ONE. 2025;20(4):e0320184. 40203229 10.1371/journal.pone.0320184 PMC11981659

[4] Collaborators GBDCD. Global burden of 292 causes of death in 204 countries and territories and 660 subnational locations, 1990–2023: a systematic analysis for the Global Burden of Disease Study 2023. Lancet. 2025;406(10513):1811–72. 41092928 10.1016/S0140-6736(25)01917-8 PMC12535838

[5] Thomson PJ, McCaul JA, Ridout F, Hutchison IL. To treat… or not to treat? Clinicians’ views on the management of oral potentially malignant disorders. Br J Oral Maxillofac Surg. 2015;53(10):1027–31. 26471841 10.1016/j.bjoms.2015.08.263

[6] Jin LJ, Lamster IB, Greenspan JS, Pitts NB, Scully C, Warnakulasuriya S. Global burden of oral diseases: emerging concepts, management and interplay with systemic health. Oral Dis. 2016;22(7):609–19. 26704694 10.1111/odi.12428

[7] Epstein JB, Huber MA. The benefit and risk of screening for oral potentially malignant epithelial lesions and squamous cell carcinoma. Oral Surg Oral Med Oral Pathol Oral Radiol. 2015;120(5):537–40. 26453381 10.1016/j.oooo.2015.07.036

[8] Scully C. Challenges in predicting which oral mucosal potentially malignant disease will progress to neoplasia. Oral Dis. 2014;20(1):1–5. 24320967 10.1111/odi.12208

[9] Mehrotra R, Gupta DK. Exciting new advances in oral cancer diagnosis: avenues to early detection. Head Neck Oncol. 2011;3:33. 21798030 10.1186/1758-3284-3-33 PMC3170277

[10] Warnakulasuriya S. Oral potentially malignant disorders: A comprehensive review on clinical aspects and management. Oral Oncol. 2020;102:104550. 31981993 10.1016/j.oraloncology.2019.104550

[11] Mello FW, Miguel AFP, Dutra KL, Porporatti AL, Warnakulasuriya S, Guerra ENS, Rivero ERC. Prevalence of oral potentially malignant disorders: a systematic review and meta-analysis. J Oral Pathol Med. 2018;47(7):633–40. 29738071 10.1111/jop.12726

[12] Iocca O, Sollecito TP, Alawi F, Weinstein GS, Newman JG, De Virgilio A, Di Maio P, Spriano G, Pardinas Lopez S, Shanti RM. Potentially malignant disorders of the oral cavity and oral dysplasia: a systematic review and meta-analysis of malignant transformation rate by subtype. Head Neck. 2020;42(3):539–55. 31803979 10.1002/hed.26006

[13] Walsh T, Macey R, Kerr AR, Lingen MW, Ogden GR, Warnakulasuriya S. Diagnostic tests for oral cancer and potentially malignant disorders in patients presenting with clinically evident lesions. Cochrane database Syst reviews (Online). 2021;7(7):CD010276. 10.1002/14651858.CD010276.pub3 PMC8407012 34282854

[14] Odell E, Kujan O, Warnakulasuriya S, Sloan P. Oral epithelial dysplasia: Recognition, grading and clinical significance. Oral Dis. 2021;27(8):1947–76. 34418233 10.1111/odi.13993

[15] Lingen MW, Kalmar JR, Karrison T, Speight PM. Critical evaluation of diagnostic aids for the detection of oral cancer. Oral Oncol. 2008;44(1):10–22. 17825602 10.1016/j.oraloncology.2007.06.011 PMC2424250

[16] Scully C, Bagan JV, Hopper C, Epstein JB. Oral cancer: current and future diagnostic techniques. Am J Dent. 2008;21(4):199–209. 18795514

[17] Gonzalez-Ruiz I, Ramos-Garcia P, Ruiz-Avila I, Gonzalez-Moles MA. Early diagnosis of oral cancer: a complex polyhedral problem with a difficult solution. Cancers (Basel). 2023;15(13). 10.3390/cancers15133270 PMC10340032 37444379

[18] McCullough MJ, Prasad G, Farah CS. Oral mucosal malignancy and potentially malignant lesions: an update on the epidemiology, risk factors, diagnosis and management. Aust Dent J. 2010;55(Suppl 1):61–5. 20553246 10.1111/j.1834-7819.2010.01200.x

[19] Mehanna HM, Rattay T, Smith J, McConkey CC. Treatment and follow-up of oral dysplasia - a systematic review and meta-analysis. Head Neck. 2009;31(12):1600–9. 19455705 10.1002/hed.21131

[20] Hanna TP, King WD, Thibodeau S, Jalink M, Paulin GA, Harvey-Jones E, O’Sullivan DE, Booth CM, Sullivan R, Aggarwal A. Mortality due to cancer treatment delay: systematic review and meta-analysis. BMJ. 2020;371:m4087. 33148535 10.1136/bmj.m4087 PMC7610021

[21] Teh MT, Hutchison IL, Costea DE, Neppelberg E, Liavaag PG, Purdie K, Harwood C, Wan H, Odell EW, Hackshaw A, et al. Exploiting FOXM1-orchestrated molecular network for early squamous cell carcinoma diagnosis and prognosis. Int J Cancer. 2013;132(9):2095–106. 23034676 10.1002/ijc.27886

[22] Ma H, Dai H, Duan X, Tang Z, Liu R, Sun K, Zhou K, Chen H, Xiang H, Wang J, et al. Independent evaluation of a FOXM1-based quantitative malignancy diagnostic system (qMIDS) on head and neck squamous cell carcinomas. Oncotarget. 2016;7(34):54555–63. 27409343 10.18632/oncotarget.10512 PMC5342363

[23] Teh MT, Ma H, Liang YY, Solomon MC, Chaurasia A, Patil R, Tekade SA, Mishra D, Qadir F, Yeung JS, et al. Molecular Signatures of Tumour and Its Microenvironment for Precise Quantitative Diagnosis of Oral Squamous Cell Carcinoma: An International Multi-Cohort Diagnostic Validation Study. Cancers (Basel). 2022;14(6):1389. 35326543 10.3390/cancers14061389 PMC8945999

[24] Gates JC, Edwards H, Villa A, Purdy N, Troka M, Varela P, Self Q, Liu Y, Dundar Y, Brooks PJ, et al. Biopsy for Suspicious Oral Lesions: A Review From the American Head and Neck Society-Cancer Prevention Service. Head Neck. 2026;48(3):884–92. 41466514 10.1002/hed.70148 PMC12891757

[25] Idrees M, Farah CS, Sloan P, Kujan O. Oral brush biopsy using liquid-based cytology is a reliable tool for oral cancer screening: A cost-utility analysis: Oral brush biopsy for oral cancer screening. Cancer Cytopathol. 2022;130(9):740–8. 35704619 10.1002/cncy.22599 PMC9544877

[26] Mehrotra R, Mishra S, Singh M, Singh M. The efficacy of oral brush biopsy with computer-assisted analysis in identifying precancerous and cancerous lesions. Head Neck Oncol. 2011;3:39. 21864339 10.1186/1758-3284-3-39 PMC3177776

[27] Bustin SA, Benes V, Garson JA, Hellemans J, Huggett J, Kubista M, Mueller R, Nolan T, Pfaffl MW, Shipley GL, et al. The MIQE guidelines: minimum information for publication of quantitative real-time PCR experiments. Clin Chem. 2009;55(4):611–22. 19246619 10.1373/clinchem.2008.112797

[28] Zhao S, Fernald RD. Comprehensive algorithm for quantitative real-time polymerase chain reaction. J Comput Biol. 2005;12(8):1047–64. 16241897 10.1089/cmb.2005.12.1047 PMC2716216

[29] Liang Y, Mo Z, Teh MT. A multigene signature for prognostic stratification of nasopharyngeal carcinoma. Cancers (Basel). 2026;18(8). 10.3390/cancers18081197 PMC13114893 42073524

[30] Robin X, Turck N, Hainard A, Tiberti N, Lisacek F, Sanchez JC, Muller M. pROC: an open-source package for R and S + to analyze and compare ROC curves. BMC Bioinformatics. 2011;12:77. 21414208 10.1186/1471-2105-12-77 PMC3068975

[31] Schwartz A, Millam G, Investigators UL. A web-based library consult service for evidence-based medicine: Technical development. BMC Med Inf Decis Mak. 2006;6:16. 10.1186/1472-6947-6-16 PMC1484475 16542453

[32] Juul N, Szallasi Z, Eklund AC, Li Q, Burrell RA, Gerlinger M, Valero V, Andreopoulou E, Esteva FJ, Symmans WF, et al. Assessment of an RNA interference screen-derived mitotic and ceramide pathway metagene as a predictor of response to neoadjuvant paclitaxel for primary triple-negative breast cancer: a retrospective analysis of five clinical trials. Lancet Oncol. 2010;11(4):358–65. 20189874 10.1016/S1470-2045(10)70018-8

[33] Appiah Adu-Gyamfi E, Tanam Djankpa F, Nelson W, Czika A, Kumar Sah S, Lamptey J, Ding YB, Wang YX. Activin and inhibin signaling: From regulation of physiology to involvement in the pathology of the female reproductive system. Cytokine. 2020;133:155105. 32438278 10.1016/j.cyto.2020.155105

[34] Bhattarai BP, Singh AK, Singh RP, Chaulagain R, Soland TM, Hasseus B, Sapkota D. Recurrence in oral leukoplakia: A systematic review and meta-analysis. J Dent Res. 2024;103:220345241266519. 10.1177/00220345241266519 PMC11504345 39290142

[35] Cirillo N. Precursor lesions, overdiagnosis, and oral cancer: a critical review. Cancers (Basel). 2024;16(8). 10.3390/cancers16081550 PMC11048740 38672632

[36] Braakhuis BJ, Tabor MP, Kummer JA, Leemans CR, Brakenhoff RH. A genetic explanation of Slaughter’s concept of field cancerization: evidence and clinical implications. Cancer Res. 2003;63(8):1727–30. 12702551

[37] Khammanivong A, Sorenson BS, Ross KF, Dickerson EB, Hasina R, Lingen MW, Herzberg MC. Involvement of calprotectin (S100A8/A9) in molecular pathways associated with HNSCC. Oncotarget. 2016;7(12):14029–47. 26883112 10.18632/oncotarget.7373 PMC4924696

[38] Chang WM, Lin YF, Su CY, Peng HY, Chang YC, Lai TC, Wu GH, Hsu YM, Chi LH, Hsiao JR, et al. Dysregulation of RUNX2/Activin-A Axis upon miR-376c Downregulation Promotes Lymph Node Metastasis in Head and Neck Squamous Cell Carcinoma. Cancer Res. 2016;76(24):7140–50. 27760788 10.1158/0008-5472.CAN-16-1188

[39] Sapkota D, Bruland O, Parajuli H, Osman TA, Teh MT, Johannessen AC, Costea DE. S100A16 promotes differentiation and contributes to a less aggressive tumor phenotype in oral squamous cell carcinoma. BMC Cancer. 2015;15:631. 26353754 10.1186/s12885-015-1622-1 PMC4564982

[40] Khera N, Rajkumar AS, Abdulkader MAK, Liu Z, Ma H, Waseem A, Teh MT. Identification of multidrug chemoresistant genes in head and neck squamous cell carcinoma cells. Mol Cancer. 2023;22(1):146. 37667354 10.1186/s12943-023-01846-3 PMC10476423

[41] Basnet S, Vallenari EM, Maharjan U, Sharma S, Schreurs O, Sapkota D. An update on S100A16 in human cancer. Biomolecules. 2023;13(7). 10.3390/biom13071070 PMC10377057 37509106

[42] Williams C, Byrne R, Holden D, Sherman I, Srinivasan VR. Two-week referrals for suspected head and neck cancer: two cycles of audit, 10 years apart, in a district general hospital. J Laryngol Otol. 2014;128(8):720–4. 25051340 10.1017/S0022215114001406

[43] Gao C, Qin C, Freeman S, Oskooee N, Hughes J. Two week wait referral criteria - heading in the right direction? J Laryngol Otol. 2019;133(8):704–12. 31370911 10.1017/S002221511900149X

[44] Scott SE, Oakley R, Moller H, Warburton F. Tracking cancer occurrence in the 5 years after referral for suspected head and neck cancer. Oral Oncol. 2020;109:104955. 32858416 10.1016/j.oraloncology.2020.104955

[45] Walsh T, Warnakulasuriya S, Lingen MW, Kerr AR, Ogden GR, Glenny AM, Macey R. Clinical assessment for the detection of oral cavity cancer and potentially malignant disorders in apparently healthy adults. Cochrane database Syst reviews (Online). 2021;12(12):CD010173. 10.1002/14651858.CD010173.pub3 PMC8664456 34891214

[46] Lingen MW, Tampi MP, Urquhart O, Abt E, Agrawal N, Chaturvedi AK, Cohen E, D’Souza G, Gurenlian J, Kalmar JR, et al. Adjuncts for the evaluation of potentially malignant disorders in the oral cavity: Diagnostic test accuracy systematic review and meta-analysis-a report of the American Dental Association. J Am Dent Assoc (1939). 2017;148(11):797–813. e752. 10.1016/j.adaj.2017.08.045 PMC7366378 29080605

[47] Lau J, O G, Warnakulasuriya S, Balasubramaniam R, Frydrych A, Kujan O. Adjunctive aids for the detection of oral squamous cell carcinoma and oral potentially malignant disorders: A systematic review of systematic reviews. Jpn Dent Sci Rev. 2024;60:53–72. 38283580 10.1016/j.jdsr.2023.12.004 PMC10821377

[48] Coletta RD, Yeudall WA, Salo T. Current trends on prevalence, risk factors and prevention of oral cancer. Front Oral Health. 2024;5:1505833. 39606098 10.3389/froh.2024.1505833 PMC11599248

[49] Mardian Y, Kosasih H, Karyana M, Neal A, Lau CY. Review of Current COVID-19 Diagnostics and Opportunities for Further Development. Front Med (Lausanne). 2021;8:615099. 34026773 10.3389/fmed.2021.615099 PMC8138031

