# Supplementary material for: INHBA–S100A16 dysregulation enables a non-invasive molecular stratification platform for rapid detection of oral squamous cell carcinoma: results from a large diagnostic case-control study
Source: Biomark Res. 2026 Jun 23;14:76. doi: 10.1186/s40364-026-00963-7 (PMC13335036; doi:10.1186/s40364-026-00963-7)
Supplement: Supplementary file 1 — Supplementary Material 1 [file 40364_2026_963_MOESM1_ESM.pdf]

INHBA–S100A16 Dysregulation Enables a Non-Invasive Molecular Stratification Platform for Rapid Detection of Oral Squamous Cell Carcinoma: Results from a Large Diagnostic Case-Control Study

Muy-Teck Teh, PhD[1*], Ranjitkumar Patil, MDS[2], Satyajit Ashok Tekade, MDS[3], Deepika Mishra, MDS[4], Akhilanand Chaurasia, MDS[2] and Ahmad Waseem, PhD[1]

[1]Centre for Oral Immunobiology & Regenerative Medicine, Institute of Dentistry, Barts & The London School of Medicine and Dentistry, Queen Mary University of London, England, United Kingdom.

[2]Department of Oral Medicine & Radiology, King George's Medical University, Lucknow, Uttar Pradesh, India.

[3]Department of Oral & Maxillofacial Pathology, Modern Dental College & Research Centre, Indore, Madhya Pradesh, India.

[4]Department of Oral Pathology & Microbiology, Centre of Dental Education & Research, All India Institute of Medical Sciences, New Delhi, India

***Corresponding Author:**
Prof. Muy-Teck Teh, BSc PhD.
Professor of Molecular Oral Oncology,
Centre for Oral Immunobiology and Regenerative Medicine,
Barts and The London School of Medicine and Dentistry,
Queen Mary University of London,
The Blizard Building,
4, Newark Street,
London E1 2AT
United Kingdom.
Tel: +44 (0) 20 7882 7140
Fax: +44 (0) 20 7882 7137
Email: m.t.teh@qmul.ac.uk
ORCID iD: orcid.org/0000-0002-7725-8355

Running title: qMIDS: A Non-Invasive Molecular Stratification Platform for Rapid OSCC Detection

Supplementary Figures

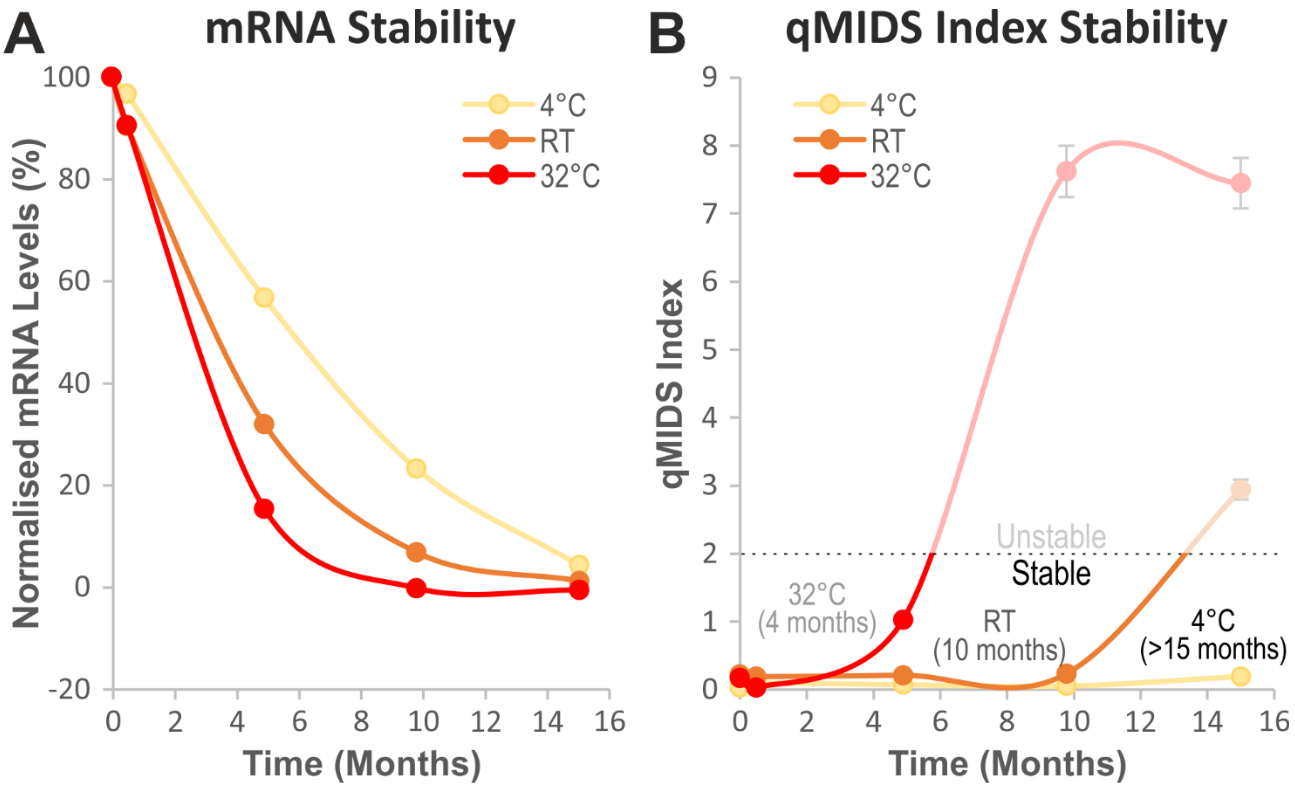

Figure S1. Temperature and time dependant mRNA and qMIDS Index stability profiles in oral brush biopsy sample over a 15-month period. **A**, An oral brush biopsy sample from a healthy volunteer (immersed in lysis buffer) was subdivided into 3 aliquots and each was kept at either 4°C (in a fridge), room temperature (RT: 18-25°C; on a lab bench) or 32°C (in a dry heat incubator). Two reference genes (*YAP1* and *POLR2A*) were measured using RT-qPCR at indicated time points (0, 15 days, 5, 10 or 15 months). Each data point represents a geometric mean ± SEM (n=4) of the two reference genes. Small SEM error bars may be obscured within the data symbols. **B**, The same samples in panel A were subjected to qMIDS^{V2} assay to obtain qMIDS index at each temperature/time incubation. A healthy oral mucosa sample is expected to produce qMIDS index of <1. Poor mRNA quality may lead to aberrant increase in qMIDS index above 2 for a normal oral mucosa sample. In this experiment, qMIDS^{V2} index remained stable throughout the 15-month period when kept at 4°C. Despite apparent mRNA degradation from 5 months onwards shown in D, demonstrating the robustness of qMIDS Index to partial mRNA degradation. For sample kept at room temperature, qMIDS index was stable for at least 10 months. For sample kept at 32°C, qMIDS index became unstable after 5 months.

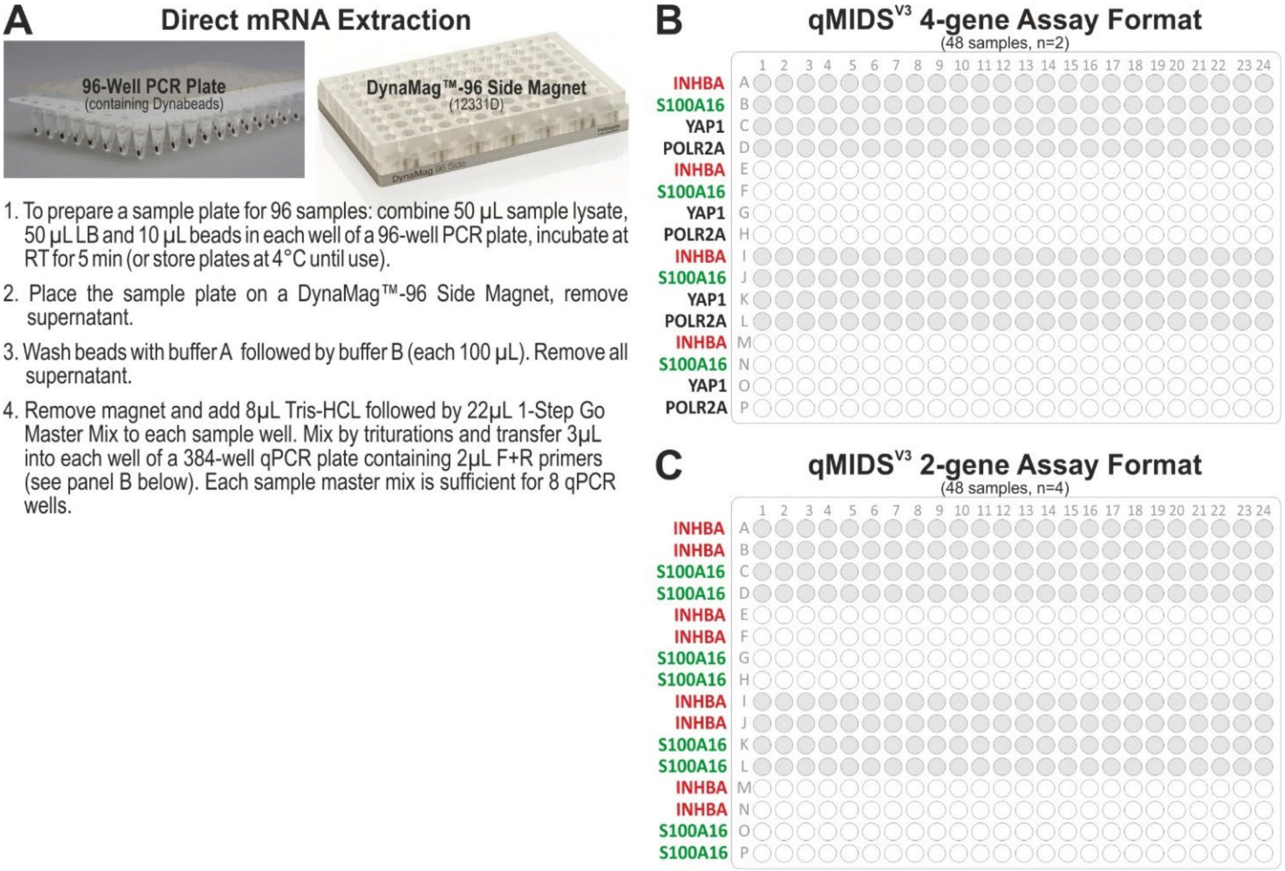

Figure S2. High-throughput mRNA purification and qMIDS^{V3} 4-gene and 2-gene assay formats. A, High-throughput mRNA direct purification from brush biopsy sample lysates using DynaMag^{TM} 96 Side Magnet (#12331D, ThermoFisher) in batches of 96 samples per extraction according to steps listed within the panel. B, qMIDS^{V3} 4-gene assay format on a 384-well qPCR plate for 48 samples with duplicate determination for each of the 4 genes as indicated. C, qMIDS^{V3} 2-gene assay measuring only INHBA and S100A16 in quadruplicates for 48 samples as indicated. In this assay, INHBA is expressed as a ratio to S100A16 in each sample.

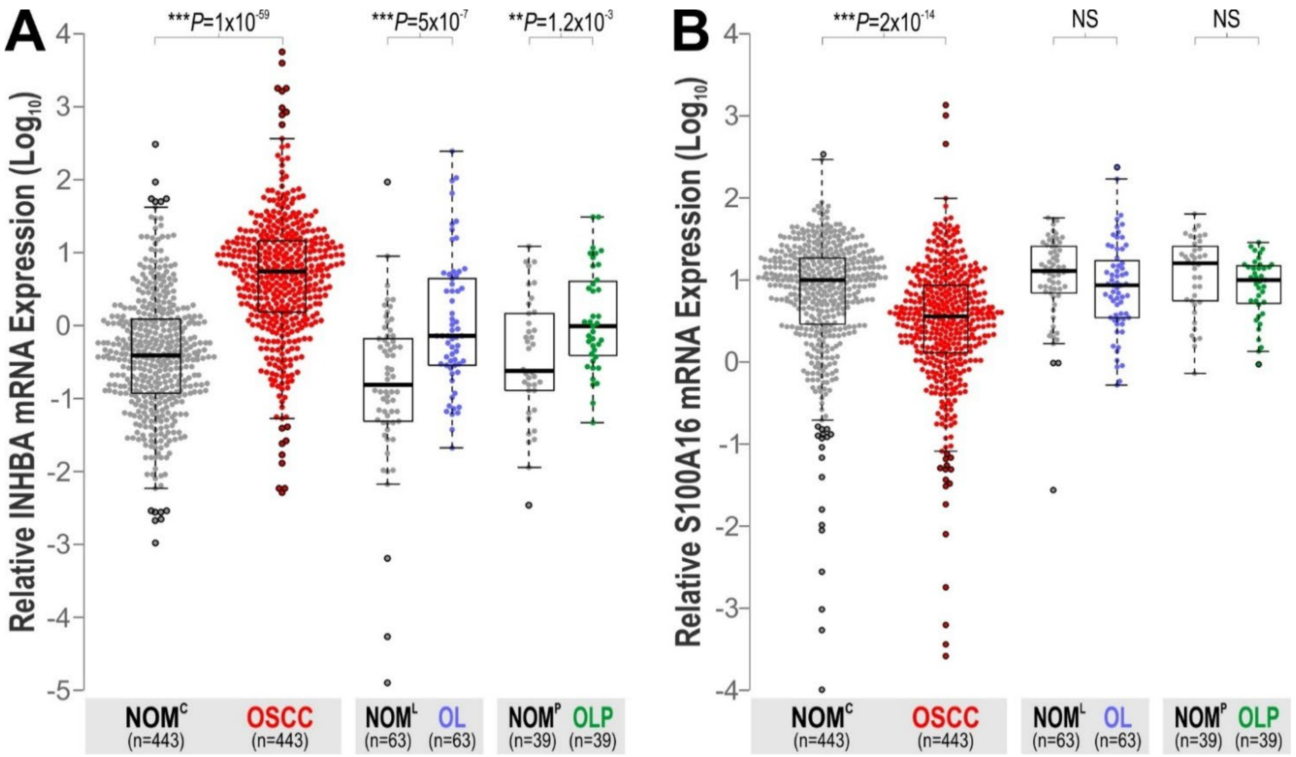

Figure S3. Relative mRNA expression levels of INHBA and S100A16 in oral brush samples of OSCC, OL and OLP. A, Beeswarm scattered box-whisker dot plots showing INHBA mRNA expression levels in paired NOM and respective lesions (OSCC, OL and OLP) as indicated. *t*-test analyses *P*-values are indicated within the figure. B, Beeswarm scattered box-whisker dot plots showing S100A16 mRNA expression levels in paired NOM and respective lesions (OSCC, OL and OLP) as indicated. Paired t-test analyses *P*-values are indicated within the figure. NS, not significant.

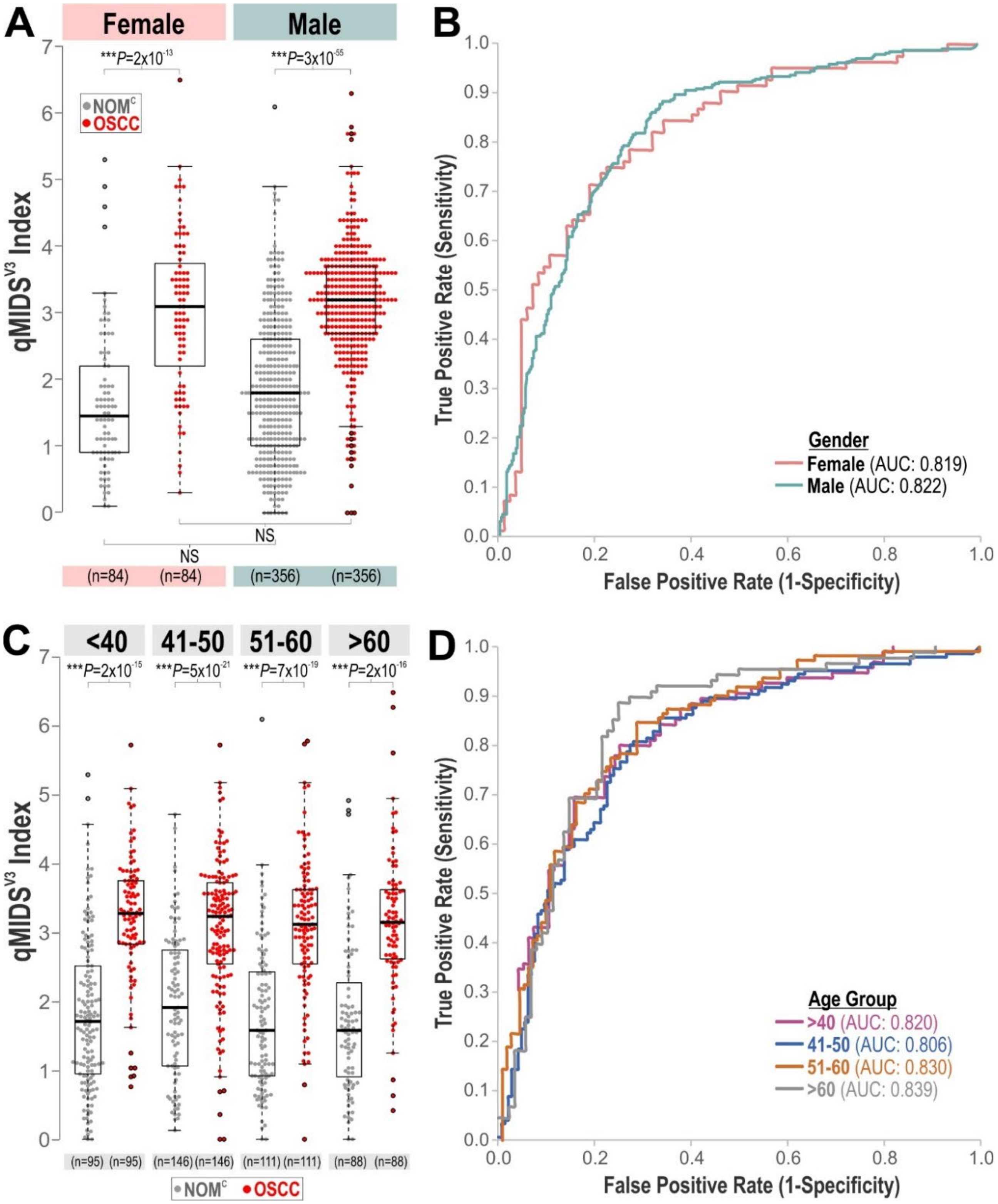

Figure S4. Sex and age differences on qMIDS^{V3} test performance on OSCC oral brush biopsy samples. A, qMIDS^{V3} Beeswarm scattered box-whisker dot plots showing data distribution for females (n=84) and males (n=356) comparing paired NOM^C and OSCC samples, respectively. Paired *t*-test analyses *P*-values are indicated within the figure. B, ROC analyses of the female and male datasets in panel A with each AUC value as indicated within the figure. C, qMIDS^{V3} Beeswarm scattered box-whisker dot plots showing data distribution across different patient age groups comparing paired NOM^C and OSCC samples as indicated. Paired *t*-test analyses *P*-values are indicated within the figure. D, ROC analyses of each age group in panel A with each AUC value as indicated within the figure.
